# Prevalence of dysphagia and eosinophilic esophagitis in patients with inflammatory bowel disease or type 2 inflammation: a cross-sectional multicenter screening study

**DOI:** 10.1177/17562848261470325

**Published:** 2026-08-28

**Authors:** Martin Bäuerle, Jurij Maurer, Jagoda Pokryszka, Gottfried Novacek, Christian Primas, Lili Kazemi-Shirazi, Stefanie Dabsch, Adrian Frick, Walter Reinisch, Clemens Dejaco, Michael Trauner, Slagjana Stoshikj, Christine Bangert, Sven Schneider, Stefan Wöhrl, Philipp Schreiner

**Affiliations:** Division of Gastroenterology and Hepatology, Department of Internal Medicine III, Medical University of Vienna, Vienna, Austria; Department of Internal Medicine I, University Hospital Wiener Neustadt, Wiener Neustadt, Austria; Danube Private University, Krems, Austria; Division of Gastroenterology and Hepatology, Department of Internal Medicine III, Medical University of Vienna, Vienna, Austria; Department of Internal Medicine IV, Klinik Landstrasse, Vienna, Austria; Division of Gastroenterology and Hepatology, Department of Internal Medicine III, Medical University of Vienna, Vienna, Austria; Division of Gastroenterology and Hepatology, Department of Internal Medicine III, Medical University of Vienna, Vienna, Austria; Division of Gastroenterology and Hepatology, Department of Internal Medicine III, Medical University of Vienna, Vienna, Austria; Division of Gastroenterology and Hepatology, Department of Internal Medicine III, Medical University of Vienna, Vienna, Austria; Division of Gastroenterology and Hepatology, Department of Internal Medicine III, Medical University of Vienna, Vienna, Austria; Division of Gastroenterology and Hepatology, Department of Internal Medicine III, Medical University of Vienna, Vienna, Austria; Division of Gastroenterology and Hepatology, Department of Internal Medicine III, Medical University of Vienna, Vienna, Austria; Division of Gastroenterology and Hepatology, Department of Internal Medicine III, Medical University of Vienna, Vienna, Austria; Division of Gastroenterology and Hepatology, Department of Internal Medicine III, Medical University of Vienna, Vienna, Austria; Division of Pulmonology, Department of Internal Medicine II, Medical University of Vienna, Vienna, Austria; Department of Dermatology, Medical University of Vienna, Vienna, Austria; Department of Otolaryngology, Head and Neck Surgery, Medical University of Vienna, Vienna, Austria; Floridsdorf Allergy Center (FAZ), Vienna, Austria; Division of Gastroenterology and Hepatology, Department of Internal Medicine III, Medical University of Vienna, Vienna 1090, Austria

**Keywords:** dysphagia, eosinophilic esophagitis, inflammatory bowel disease (IBD), type-2 inflammatory disease

## Abstract

**Background:**

Eosinophilic esophagitis (EoE) is a chronic, type-2 inflammation-driven disease causing dysphagia. Although patients with other type-2 inflammatory diseases (type-2 ID) or inflammatory bowel disease (IBD) may have an increased risk of EoE, there is limited information about the prevalence of EoE in these specific populations.

**Objectives:**

This study aims to assess the prevalence of clinically relevant dysphagia as well as underlying EoE in patients with type-2 ID or IBD.

**Design:**

This is a cross-sectional, multicenter study of patients with type-2 ID (asthma, rhinosinusitis with polyposis nasi, allergic rhinitis, atopic dermatitis, Immunoglobulin E (IgE)-mediated food allergies) or IBD from the Medical University of Vienna and Floridsdorf Allergy Center.

**Methods:**

Patients were screened for esophageal dysphagia using the Brief Esophageal Dysphagia Questionnaire (BEDQ). Relevant dysphagia was defined as a BEDQ score ⩾4, or having a history of bolus impaction or emergency department visit due to dysphagia within the past year. Endoscopy with esophageal biopsies was offered to all patients with relevant dysphagia to screen for EoE.

**Results:**

A total of 687 patients (45.9% female, median age 45 years, 53.9% with IBD, 48.5% with a type 2 ID) were screened. Overall, 8.9% (*n* = 61) reported relevant dysphagia. Endoscopy with biopsies was performed in 22 patients, and 18 patients had undergone endoscopy the year prior to the study. In total, 6 newly diagnosed cases of EoE were observed, while 7 patients had a known history of EoE, resulting in an overall prevalence of 13 patients (1.9%). Patients with any type-2 ID had numerically more EoE (2.7%) than patients with IBD (1.9%).

**Conclusion:**

While the prevalence of dysphagia among patients with type-2 ID and IBD may not exceed that of the general population, the prevalence of EoE in these populations may be higher than previously recognized.

## Plain language summary

Prevalence of dysphagia and eosinophilic esophagitis in patients with inflammatory bowel disease or type 2 inflammation—a cross-sectional multicenter screening study

Eosinophilic esophagitis (EoE) is a chronic, type-2 inflammation-driven disease causing dysphagia. Although patients with other type-2 inflammatory diseases or inflammatory bowel disease may have an increased risk of EoE, there is limited information about the real prevalence in these specific populations. We therefore screened 687 patients with type-2 inflammatory diseases (asthma, rhinosinusitis with polyposis nasi, allergic rhinitis, atopic dermatitis, IgE-mediated food allergies) or inflammatory bowel disease for esophageal dysphagia using a validated questionnaire. Therefore, 61 patients (8.9%) with clinically relevant dysphagia were offered endoscopy with esophageal biopsies. In total, six newly diagnosed cases of EoE were observed, while seven patients had a known history of EoE, resulting in an overall prevalence of 13 patients (1.9%).

## Introduction

Eosinophilic esophagitis (EoE) is a chronic, type-2 inflammation-driven disease of the esophagus causing esophageal dysfunction. Dysphagia is the hallmark symptom of EoE, and esophageal food impaction is a consequence of esophageal inflammation with or without fibrotic remodeling. The diagnosis of EoE is established based on the presence of esophageal dysfunction, an esophageal biopsy showing ⩾15 eosinophils per high-power field (eos/hpf), and the exclusion of other disorders that could cause esophageal eosinophilia.^1,2^ A recent meta-analysis demonstrated a continuing increasing global incidence and prevalence of EoE with up to 5.31 cases per 100,000 inhabitant-years and 40.04 cases per 100,000 inhabitants, respectively.^3^ Since a recent population-based analysis showed an even higher prevalence of 128/100,000 adult inhabitants in Sweden, it may be assumed that these numbers of known EoE cases represent merely the tip of the iceberg.^4^

There exists a strong association between EoE and other atopic diseases such as asthma, allergic rhinitis, atopic dermatitis, and IgE-mediated food allergies and up to 80% of patients with EoE have a concomitant allergic disease.^5–9^ These findings suggest a shared immunological and pathophysiological Th2-mediated pathway.^9^ This leads to epithelial barrier dysfunction and alarmin release like TSLP, IL-25, or IL-33 in response to epithelial damage. This in turn activates Th2 cells and innate lymphoid cells type 2, which produce key inflammatory type 2 cytokines such as IL-4, IL-5, and IL-13. The production of these cytokines further promotes Th2-cell differentiation, IgE class switching, eosinophil inflammation, mucus production, epithelial remodeling, fibrosis, and barrier damage.^10,11^

Although evidence is limited, a positive association between EoE and inflammatory bowel disease (IBD) might be existent.^12–15^ The exact mechanism underlying these overlapping diseases is elusive but may be explained by similar helper T-cell-mediated inflammation of the luminal gastrointestinal tract.^16^

Since EoE is a chronic, progressive disorder associated with an increasing risk of strictures in untreated patients, an early diagnosis is of utmost importance. However, despite efforts in awareness and education, diagnostic delay has not been improved in the last decades.^17^

In this multicenter study, we aimed to screen patients with type-2 inflammatory disease (type-2 ID) and IBD for dysphagia and EoE.

## Patients and methods

We performed a cross-sectional questionnaire-based survey of adults with IBD or a type-2 ID. Patients were recruited at different departments of the Medical University of Vienna, Austria (Gastroenterology/Hepatology, Dermatology, Pulmonology, Otorhinolaryngology) and at the Floridsdorf Allergy Center (FAZ), Vienna, Austria. The questionnaires were collected between June 2024 and January 2025, and consecutive endoscopy was performed from June 2024 to November 2025. Inclusion criteria were all consenting patients with an age ⩾18 years attending routine outpatient visits with a diagnosis of asthma, atopic dermatitis, chronic rhinosinusitis with nasal polyps (CRSwNP), IgE-mediated food allergy, allergic rhinitis, or IBD, independent of their current treatment status for the underlying type 2 inflammatory disease and were selected consecutively. Only participants with a completed questionnaire were included. Exclusion criteria were age <18 years, pregnant patients, and inability to provide informed consent. Patients were approached during their scheduled visits and invited to participate in dysphagia screening.

## Screening procedure

Dysphagia was assessed using the validated version of the Brief Esophageal Dysphagia Questionnaire (BEDQ).^18^ Translation of the BEDQ into German was performed in accordance with the World Health Organization guidelines for translation and adaptation of instruments (forward and backward translation). In brief, the BEDQ is a questionnaire with five questions about the frequency and three questions about the severity of dysphagia in the past 14 days from 0 (no symptoms) to 40 and about complications due to dysphagia (within the past 12 months whether food stuck in the throat or esophagus for a period lasting longer than 30 min or a visit to the emergency room because of food stuck in the throat or esophagus). The questionnaire was administered by trained interviewers during routine clinic visits. As no validated cut-off for the BEDQ is available, relevant dysphagia (moderate and severe dysphagia) was defined with a score equal or more than 4 to have a sensitivity of 90%).^18^

- 0: no dysphagia
- 1–3: mild dysphagia
- 4–9: moderate dysphagia
- ⩾10: severe dysphagia

Patients with moderate to severe dysphagia, defined as a BEDQ score from 4 to 9 (moderate) or ⩾10 (severe), or a complication due to dysphagia were offered an esophagogastroduodenoscopy to diagnose/exclude EoE if they have not had a Esophagogastroduodenoscopy (EGD) with biopsies within the last year. EGD was performed by an EoE specialist according to the current guidelines, which require six biopsies from a minimum of two different locations in the distal and proximal esophagus also in the absence of macroscopic abnormalities, as well as targeted biopsies from visible lesions. Diagnosis of EoE or EoE-variants was established by known criteria.^19,20^

Potential confounders as concomitant medical therapy with dupilumab, benralizumab, tezepelumab were obtained through the questionnaire. Peripheral blood samples were collected and analyzed at the respective institutions. Absolute eosinophil count, eosinophilic cationic protein (ECP), and total IgE levels were determined using standard assays.

### Statistical analysis

Data were collected using Jamovi^®^ [The jamovi project (2024), version 2.7.11.0] and Microsoft Excel^®^ [Microsoft Corporation (Redmond, Washington, USA), version 16.89.1]. Categorical values are presented as fractions of total, continuous ones—as medians with interquartile ranges or means with standard deviations.

A regression model was set up to evaluate which factors are associated with high BEDQ score. To account for the variability between the centers’ populations, a random effect (1|center) was introduced.^21^ High score was chosen as a dependent binary outcome. High score was defined as BEDQ >4 or within the past 12 months whether food stuck in the throat or esophagus for a period lasting longer than 30 min or a visit to the emergency room because of food stuck in the throat or esophagus). Following independent variables were tested in a backward selection process: treatment with dupilumab or tezepelumab (for coexisting type II immunity disorders), age, sex, known EoE, concomitant IBD, concomitant asthma. Other comorbidities were not included in the model as there were too few observations. The choice of the independent variables was based on the literature search.^22,23^ Finally, the most suitable model was chosen after comparing Akaike’s Information Criterion.^24^ The significance of the *p*-value was set at 0.05. The statistical analysis was performed in (R Core Team, v. 4.4.3).^25^

For the dysphagia evaluation, only participants with completed questionnaires were included, while patients with missing questionnaires were excluded. For missing laboratory measurements or endoscopic examinations, analyses were only performed with available data.

As this was a pilot study, no sample calculations were conducted, and all patients with a scheduled follow-up who agreed to participate were included.

The authors used ChatGPT (OpenAI) to support language refinement and text structuring. All final content was critically reviewed by the authors.^26^

### Ethics approval

Approval was obtained from the ethics committee of the Medical University of Vienna (EK 1280/2024), and written informed consent was obtained from all participants.

## Results

A total of 687 patients (45.9% female, median age 45 years) with type-2 IDs and IBD were screened for dysphagia using the validated German version of the BEDQ.

Patient characteristics are shown in Table 1. 53.9% of all participants had a diagnosis of IBD, and 48.5% had a type 2-ID (24.2% allergic rhinitis, 20.8% asthma, 11.2% atopic dermatitis, 10.8% chronic rhinosinusitis with nasal polyps (CRSwNP) and 6.0% IgE-mediated food allergy) with multiple diagnoses possible. Overall, 72 participants (10.5%) had a treatment with dupilumab and 14 participants (2.0%) with Tezepelumab. Baseline laboratory measurements including serum-IgE levels, absolute eosinophil counts, and ECP are also shown in Table 1.

**Table 1.** Patient characteristics, dysphagia evaluation, and patients’ allergy panel results.

Patients answered the BEDQ	Total (*n* = 687)	IBD (*n* = 370)	Asthma (*n* = 143)	Atopic dermatitis (*n* = 77)	IgE-mediated food allergy (*n* = 41)	Allergic rhinitis (*n* = 166)	Chronic rhinosinusitis with nasal polyps (*n* = 74)
Age in years (median, IQR)	45 (26.0)	44.0 (25.8)	52 (21.5)	41 (21.0)	39 (18.0)	38.5 (25.0)	53.0 (25.0)
Sex (w in %)	315 (45.9%)	151 (40.8%)	82 (57.3%)	33 (42.9%)	18 (43.9%)	73 (44.0%)	33 (44.6%)
Other type 2 inflammatory disease or IBD							
IBD	370 (53.9%)		8 (5.6%)	7 (9.1%)	7 (17.1%)	14 (8.4%)	6 (8.1%)
Asthma	143 (20.8%)	8 (2.2%)		13 (16.9%)	13 (31.7%)	46 (27.7%)	44 (59.5%)
Atopic dermatitis	77 (11.2%)	7 (1.9%)	13 (9.1%)		9 (22.0%)	36 (21.7%)	3 (4.1%)
IgE-mediated food allergy	41 (6.0%)	7 (1.9%)	13 (9.1%)	9 (11.7%)		27 (16.3%)	8 (10.8%)
Allergic rhinitis	166 (24.2%)	14 (3.8%)	46 (32.2%)	36 (46.8%)	27 (65.9%)		20 (27.0%)
Chronic rhinosinusitis with polyposis nasi	74 (10.8%)	6 (1.6%)	44 (30.8%)	3 (3.9%)	8 (19.5%)	20 (12.0%)	
Oral allergy syndrome (%)	38 (5.5%)	20 (5.4%)	11 (7.7%)	8 (10.4%)	6 (14.6%)	15 (9.0%)	5 (6.8%)
Dupilumab treatment (%)	72 (10.5%)	1 (0.3%)	26 (18.2%)	28 (36.4%)	4 (9.8%)	21 (12.7%)	35 (47.3%)
Tezepelumab treatment (%)	14 (2.0%)	1 (0.3%)	13 (9.1%)	0 (0%)	2 (4.9%)	2 (1.2%)	1 (1.4%)
Known EoE	7 (1.0%)	2 (0.5%)	1 (0.7%)	0 (0%)	2 (4.9%)	3 (1.8%)	0 (0%)
BEDQ							
0	558 (81.2%)	326 (88.1%)	88 (61.5%)	68 (88.3%)	28 (68.3%)	130 (78.3%)	47 (63.5%)
1–3	73 (10.6%)	28 (7.6%)	25 (17.5%)	6 (7.8%)	7 (17.1%)	19 (11.4%)	18 (24.3%)
4–9	33 (4.8%)	11 (3.0%)	15 (10.5%)	3 (3.9%)	3 (7.3%)	12 (7.2%)	7 (9.5%)
⩾10	23 (3.3%)	5 (1.4%)	15 (10.5%)	0 (0%)	3 (7.3%)	5 (3.0%)	2 (2.7%)
Food bolus impaction in the last 12 months	16 (2.3%)	6 (1.6%)	8 (5.6%)	0 (0%)	1 (2.4%)	7 (4.2%)	4 (5.4%)
IgE (median, IQR)	54.8 (144) (*n* = 490/687)	32.5 (71.8) (*n* = 204/370)	67.8 (212) (*n* = 130/143)	586 (2524) (*n* = 59/77)	130 (397) (*n* = 37/41)	110 (312) (*n* = 154/166)	50.7 (98.5) (*n* = 69/74)
Absolute eosinophils (median, IQR)	0.20 (0.20) (*n* = 569/687)	0.20 (0.20) (*n* = 362/370)	0.20 (0.30) (*n* = 135/143)	0.30 (0.30) (*n* = 66/77)	0.20 (0.25) (*n* = 23/41)	0.20 (0.30) (*n* = 74/166)	0.30 (0.40) (*n* = 72/74)
ECP (median, IQR)	33.8 (37.5) (*n* = 355/687)	30.1(27.5) (*n* = 190/370)	41.2 (41.5) (*n* = 68/143)	43.1 (43.0) (*n* = 54/77)	26.1 (44.4) (*n* = 21/41)	32.7 (42.5) (*n* = 77/166)	40.6 (46.2) (*n* = 55/74)

ECP, eosinophilic cationic protein; EoE, eosinophilic esophagitis; IBD, inflammatory bowel disease; IQR, interquartile range.

### Prevalence of dysphagia

**Figure 1.**
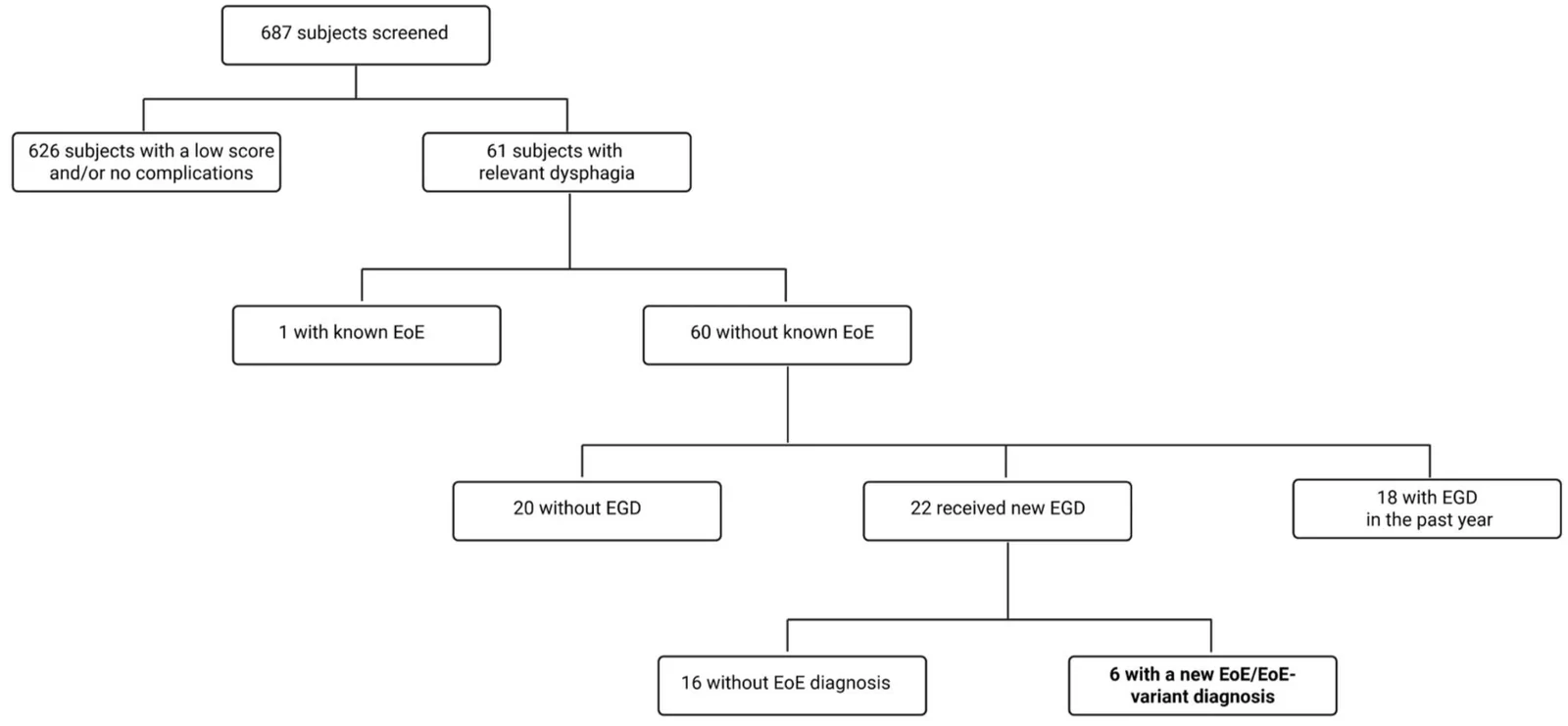
Flowchart of study population.

Six hundred thirty-one patients (91.8%) reported no or mild dysphagia (BEDQ-score 0–3), 56 patients (8.2%) reported moderate to severe dysphagia (BEDQ-score ⩾4) and 16 patients (2.3%) had a food bolus impaction in the past 12 months including 5 patients with no or mild dysphagia (Figure 1).

Overall, 8.9% (*n* = 61) reported either moderate to severe dysphagia and/or complication due to dysphagia (relevant dysphagia). The difference in baseline characteristics between patients with relevant dysphagia versus patients without relevant dysphagia is described in Table 2. While both the relative and absolute number of patients with no/mild dysphagia had IBD as the most common diagnosis, asthma was the most frequent diagnosis in patients with moderate/severe dysphagia or with food bolus impaction. Furthermore, serum-IgE and ECP levels were higher in the relevant dysphagia group than in the no/mild dysphagia group, whereas absolute eosinophils count was similar between the groups.

**Table 2.** Comparison of the patients with no-to-mild and moderate-to-severe dysphagia.

Patients answered the BEDQ	No/mild dysphagia without bolus impaction (*n* = 626)	Moderate/severe dysphagia or bolus impaction (*n* = 61)
Age in years (median, IQR)	44.5 (25.0)	53.0 (22.0)
Sex (w in %)	284 (45.4)	31 (50.8)
Inflammatory disease, no. (%)		
IBD	351 (56.1)	19 (31.1)
Asthma	113 (18.1)	30 (49.2)
Atopic dermatitis	74 (11.8)	3 (4.9)
IgE-mediated food intolerance	35 (5.6)	6 (9.8)
Allergic rhinitis	147 (23.5)	19 (31.1)
Chronic rhinosinusitis with polyposis nasi	64 (10.2)	10 (16.4)
Oral allergy syndrome (%)	34 (5.4)	4 (6.6)
Dupilumab treatment (%)	66 (10.5)	6 (9.8)
Tezepelumab treatment (%)	10 (1.6)	4 (6.6)
IgE (median, IQR)	52.8 (142)	63.3 (173)
Absolute eosinophils (median, IQR)	0.2 (0.2)	0.2 (0.2)
ECP (median, IQR)	32.6 (37.4)	41.5 (33.8)

ECP, eosinophilic cationic protein; IBD, inflammatory bowel disease; IQR, interquartile range.

The final model describing the association between patient’s characteristics and probability of relevant dysphagia included centers as random effect. The fixed effects (age and presence of asthma) explained only 8.4% of the model’s variance (marginal *R*^2^), which indicates limited effect of the predictors on the relevant dysphagia. Both fixed and random effects explained 15% of the variance (conditional *R*^2^; see Table 3).

**Table 3.** Logistic regression analysis of factors associated with high dysphagia scores.

Predictors	High_score		
	Odds ratios	CI	*p*
(Intercept)	0.03	0.01–0.09	<0.001
Age	1.02	1.00–1.03	0.074
Asthma^1^	3.08	1.27–7.47	0.013

### Prevalence of EoE

EoE had been diagnosed previously in seven patients, with one of the patients also reporting relevant dysphagia in the BEDQ. Among the remaining 60 patients with relevant dysphagia, 40 had undergone or recently received an endoscopy, which ruled out EoE in most cases. However, 6 new cases of eosinophilic/lymphocytic esophagitis were diagnosed, and 20 patients refused gastroscopy mostly due to personal preference or because they could not be reached up for follow-up (see Figure 1). The mean Eosinophilic Esophagitis Endoscopic Reference Score (EREFS) score in this group was 1.83 (SD 1.72), and the mean peak eos/hpf was 51.4 (SD 32.32) with one patient having >20 lymphocytes/hpf. In total, 13 patients (38.5% female, median age 40 years) from our entire cohort were newly diagnosed or already had a diagnosis of EoE or EoE variant (1.9%). Overall, patients with IBD had a lower prevalence (1.9% (7/370)) than patients with any type-2 ID (2.7% (9/333)). Nevertheless, 53.8% (*n* = 7) of patients with a newly diagnosed EoE had an IBD and 38.5% (*n* = 5) and allergic rhinitis. As patients could have both IBD and one or more type 2 inflammatory diseases, these categories were not mutually exclusive, and the counts should not be summed. Furthermore, serum IgE levels, absolute eosinophil counts, and ECP levels were numerically higher in this cohort compared with the overall cohort (see Table 4).

**Table 4.** Subgroup analysis of patients with known or newly diagnosed EoE.

Patient with known or newly diagnosed EoE	Total (*n* = 13)
Age in years (median, IQR)	40 (17.0)
Sex (w:m)	5:8
IgE (median, IQR)	586 (1140)
Absolute eosinophils (median, IQR)	0.50 (0.325)
ECP (median, IQR)	37.0 (53.0)
IBD	7 (53.8%)
Asthma	2 (15.4%)
Atopic dermatitis	3 (23.1%)
IgE-mediated food intolerance	3 (23.1%)
Allergic rhinitis	5 (38.5%)
Chronic rhinosinusitis with polyposis nasi	0

ECP, eosinophilic cationic protein; EoE, eosinophilic esophagitis; IBD, inflammatory bowel disease; IQR, interquartile range.

## Discussion

In this cross-sectional screening study, we systematically assessed esophageal dysphagia in adults with type 2 ID and IBD using a standardized instrument. To our knowledge, this represents the first prospective screening approach in a real-world cohort aimed at detecting previously unrecognized EoE in these populations. However, a prospective study in a Swedish population cohort conducted between 1990 and 2017 followed patients with biopsy-verified EoE and found that EoE was associated with a 3.5-fold increased risk of subsequently developing IBD.^15^ Moreover, several retrospective studies including one recent study by Haber et al. indicate that patients with atopic diseases and IBD are at an increased risk of developing EoE.^6,12,13,23,27^

Following main findings emerge^1^:

First of all, the prevalence of dysphagia may be comparable between patients with type 2 ID or IBD and the general population.^2^

Dysphagia is relatively common in the general population with a prevalence between 8% and 16%.^28–30^ However, nearly all studies investigating dysphagia used a dichotomous question about difficulty of swallowing and not a validated questionnaire. Furthermore, most validated questionnaires screen for oropharyngeal and not esophageal dysphagia.^31,32^ Additionally, available questionnaires designed for esophageal dysphagia or specifically for EoE (Dysphagia Symptom Questionnaire^33^), Eosinophilic Esophagitis Symptom Activity Index,^34^ Mayo Dysphagia Questionnaire-30,^35^ are either not validated, require repeated measurements, or are not designed for a screening purpose. Consequently, we used the BEDQ as one of the validated questionnaires to screen for esophageal dysphagia.

However, our results indicate that patients with asthma are at increased risk of developing dysphagia, which may be related to the higher prevalence of reflux disease,^36^ a common cause of dysphagia,^37^ or to the increased risk of developing EoE.^27^

Secondly, with nearly 2% of newly diagnosed or known EoE patients in this cross-sectional screening study, the prevalence of EoE clearly exceeds the reported estimates for the general population in our study cohort.^3^ Given that 10.3% of all participants had a therapy with dupilumab and even 30% of patients with dysphagia but normal endoscopic and histological findings were treated with eosinophilic depleting medications as dupilumab, benralizumab, and tezepelumab, the true number of patients with EoE may be even higher. Several studies demonstrate that symptoms and histologic findings have only a modest correlation^38^ and some eosinophilic-depleting medications have rarely an effect on dysphagia symptoms despite completely depleting eosinophils in the esophageal tissue.^39,40^ Therefore, these anti-eosinophilic medications may have masked some EoE cases.

Noteworthy, dupilumab treatment was administered bi-weekly (300 mg in CRSwNP and 200 mg in atopic dermatitis) in all participants of the survey, whereas this dosing interval showed no significant symptom improvement compared to placebo in a phase III trial.^41^

Although we used a low threshold of 4 in defining relevant dysphagia to have at least a sensitivity of 90%^18^ and a specific questionnaire for esophageal questionnaire, we may have missed some patients with a mild EoE. Since the BEDQ does not include questions about potential behavioral adaptation such as slow eating or prolonged chewing, mechanisms that often exist in EoE patients,^42^ several patients with such coping mechanism could have a low score in BEDQ.

EoE is a progressive disease that may lead to fibrotic changes in untreated patients. It is estimated that every additional year of undiagnosed EoE increases the risk of a stricture by 9%. Therefore, an early diagnosis is crucial to prevent progression from an inflammatory to a fibrostenotic phenotype.^17,43^ Data from the EoE CONNECT registry showed a reduction in diagnostic delay.^44^ In contrast, a recent study from Switzerland demonstrated a persistent long diagnostic delay of ⩾10 years in a third of patients without a change in the last 30 years.^45^

Since the prevalence of EoE in our survey (1.9%) clearly exceeded estimates reported for the general adult population (approximately 0.1%), patients with type-2 ID and IBD could be regarded as high-risk group. The question arises whether a screening in such a selected patient group should be recommended and if so, how a screening for EoE should be established. According to the first screening criteria of Wilson and Jungner,^46^ the condition (EoE) should be an important health problem. Although EoE has a relevant economic impact on society with nearly 50% higher societal costs,^47^ it is not associated with a higher mortality and the rationality of a screening program is questionable. Furthermore, an EoE is defined with esophageal symptoms and with at least 15 eosinophils per high-power field in the esophageal tissue. Therefore, it needs at least a questionnaire and additionally an EGD to establish a diagnosis.^1^

In contrast, the natural history of EoE is well understood, an early diagnosis is possible, and treatment at an early stage is beneficial with a reasonable cost/benefit ratio. Hence, a questionnaire to screen for dysphagia could be considered as a first-step approach; however, its role in high-risk individuals requires further investigation.

Nearly all patients with EoE in our survey who had an established diagnosis prior to completing the questionnaire—including, interestingly, two patients from our IBD clinic—had not informed their treating physician about their previous EoE diagnosis and were not receiving EoE treatment at the time of the questionnaire. Our data underscore that education about long-term management in EoE patient needs improvement.

Our study has several limitations. First, patients were recruited from a specialized allergy center and a university hospital, which could lead to selection bias. Furthermore, dysphagia assessment relied on a questionnaire, and while validated, optimal cut-off values for relevant dysphagia in this specific population were not formally validated and remain unknown—therefore they should be interpreted with caution. Additionally, not all eligible patients underwent endoscopy, and refusal or prior non-standardized endoscopy evaluations may have led to an underestimation of EoE prevalence. With a prevalence of EoE of around 1:1000 in the general population, more than 30,000 persons with type-2 ID or IBD should be screened for an adequate statement about a correct prevalence in this specific population. However, to find at least one case of EoE with a prevalence of 1:1000 and a 95% CI, around 3000 patients should be screened. Therefore, our study is clearly underpowered for an adequate estimation of a correct prevalence, but the key message, namely the exceeding numbers of patients with EoE in our screened population, remain true. Since neither the use of systemic corticosteroids, inhaled corticosteroids nor over-the-counter medications such as proton-pump inhibitors were assessed in our study, we cannot exclude the possibility that some patients had already been treating an undiagnosed EoE. The actual number of patients with dysphagia or elevated eosinophils in the biopsies may therefore have been even higher. Furthermore, we did not have a control group (healthy population) that completed the same questionnaire. Finally, the number of EoE cases was small, limiting subgroup analyses and biomarker interpretation.

In conclusion, cross-sectional symptom-based screening with an addition of an EGD in positive findings reveals that EoE could be more prevalent in type-2 ID and IBD compared to the general population, but larger sample sizes would be necessary for a more precise statement. Routine, structured assessment of dysphagia in clinical practice in high-risk patients could be justified and may help reduce diagnostic delay of EoE.

## Supplemental Material

sj-doc-1-tag-10.1177_17562848261470325 – Supplemental material for Prevalence of dysphagia and eosinophilic esophagitis in patients with inflammatory bowel disease or type 2 inflammation: a cross-sectional multicenter screening study Supplemental material, sj-doc-1-tag-10.1177_17562848261470325 for Prevalence of dysphagia and eosinophilic esophagitis in patients with inflammatory bowel disease or type 2 inflammation: a cross-sectional multicenter screening study by Martin Bäuerle, Jurij Maurer, Jagoda Pokryszka, Gottfried Novacek, Christian Primas, Lili Kazemi-Shirazi, Stefanie Dabsch, Adrian Frick, Walter Reinisch, Clemens Dejaco, Michael Trauner, Slagjana Stoshikj, Christine Bangert, Sven Schneider, Stefan Wöhrl and Philipp Schreiner in Therapeutic Advances in Gastroenterology

## Declarations

**Ethics approval and consent to participate:** Approval was obtained from the ethics committee of the Medical University of Vienna (EK 1280/2024). Written informed consent was obtained from all participants.

**Consent for publication:** Not applicable.

**Availability of data and materials:** The dataset used during the current study is available from the corresponding author if needed.*
